# Excessive sitting at work and at home: Correlates of occupational sitting and TV viewing time in working adults

**DOI:** 10.1186/s12889-015-2243-y

**Published:** 2015-09-15

**Authors:** Nyssa T. Hadgraft, Brigid M. Lynch, Bronwyn K. Clark, Genevieve N. Healy, Neville Owen, David W. Dunstan

**Affiliations:** Baker IDI Heart and Diabetes Institute, Melbourne, Australia; School of Public Health and Preventive Medicine, Monash University, Melbourne, Australia; Cancer Epidemiology Centre, Cancer Council Victoria, Melbourne, Australia; Melbourne School of Population & Global Health, University of Melbourne, Melbourne, Australia; School of Public Health, The University of Queensland, Brisbane, Australia; Centre for Physical Activity and Nutrition Research, Deakin University, Melbourne, Australia; Department of Medicine, Monash University, Melbourne, Australia; School of Sport Science, Exercise and Health, The University of Western Australia, Perth, Australia

**Keywords:** Sedentary behaviour, Correlates, Occupational sitting, Television viewing

## Abstract

**Background:**

Recent evidence links sedentary behaviour (or too much sitting) with poorer health outcomes; many adults accumulate the majority of their daily sitting time through occupational sitting and TV viewing. To further the development and targeting of evidence-based strategies there is a need for identification of the factors associated with higher levels of these behaviours. This study examined socio-demographic and health-related correlates of occupational sitting and of combined high levels of occupational sitting/TV viewing time amongst working adults.

**Methods:**

Participants were attendees of the third wave (2011/12) of the Australian Diabetes, Obesity and Lifestyle (AusDiab) study who worked full-time (≥35 h/week; n = 1,235; 38 % women; mean ± SD age 53 ± 7 years). Logistic and multinomial logistic regression analyses were conducted (separately for women and men) to assess cross-sectional associations of self-reported occupational sitting time (categorised as high/low based on the median) and also the combination of occupational sitting time/TV viewing time (high/low for each outcome), with a number of potential socio-demographic and health-related correlates.

**Results:**

Higher levels of occupational sitting (>6 h/day) were associated with higher household income for both genders. Lower levels of occupational sitting were associated with being older (women only); and, for men only, having a blue collar occupation, having a technical/vocational educational attainment, and undertaking more leisure-time physical activity (LTPA). Attributes associated with high levels of both occupational sitting and TV viewing time included white collar occupation (men only), lower levels of LTPA (both genders), higher BMI (men), and higher energy consumption (women).

**Conclusions:**

Higher household income (both genders) and professional/managerial occupations (men only) were correlates of high occupational sitting time, relative to low occupational sitting time, while health-related factors (lower LTPA, higher BMI – men, and higher energy consumption – women) were associated with high levels of both occupational sitting and TV viewing time, relative to low occupational sitting and low TV viewing time. These findings suggest possible high-risk groups that may benefit from targeted interventions. Further research is needed on potentially modifiable environmental and social correlates of occupational sitting time, in order to inform workplace initiatives.

## Background

Evidence is accumulating on the detrimental health consequences of sedentary behaviour, or too much sitting. Amongst adults, studies have observed increased risk of cardiovascular and all-cause mortality, some cancers, type 2 diabetes and the metabolic syndrome with higher levels of sedentary behaviour [1–4]. Importantly, these detrimental associations have persisted in studies that have controlled for moderate-vigorous or leisure-time physical activity [3], suggesting the need for a dedicated public health focus on too much sitting.

In order to effectively intervene and reduce overall levels of sedentary behaviour, it is important to understand the settings in which it occurs, and the specific factors influencing high levels of sedentary behaviour in these particular settings. For many adults, the occupational setting is where a large proportion of daily sedentary time is accrued [5]. Recent studies using objective monitoring have indicated that office-based workers spend at least two-thirds of their working hours sedentary [6, 7]. With technological advances automating many previously manual tasks, sitting has become the normative posture in many workplaces [8]. This, coupled with the increasing recognition of the adverse health impacts, has led some to propose that occupational sitting should be identified as a potential hazard and treated accordingly under work health and safety laws [9].

In this context, there is the need to identify the relevant attributes of those working adults who are most sedentary, in order to strengthen the evidence base required to inform future workplace guidelines, programs and policies. Of the few studies that have examined the attributes associated with sedentary behaviour in the work environment, employment characteristics and socio-demographic attributes have been identified as individual-level correlates [10]. In particular, evidence suggests that workers in physically demanding and blue-collar occupations have been found to have lower levels of occupational sitting than those in physically undemanding and white collar jobs [11, 12]. Higher educational attainment and income [10, 13], being male, younger and having a higher BMI also appear to be attributes linked with higher levels of occupational sitting [10].

When considering working adults’ opportunities to be sedentary, occupational sitting time combined with television viewing time account for the greatest proportion of sedentary waking hours on work days [14, 15]. TV viewing is the most common leisure-time sedentary behaviour in Australia, the UK and the USA [16–18] and there is consistent evidence linking high levels of TV viewing time with adverse health outcomes including the metabolic syndrome, cardiovascular disease and premature mortality [1, 2, 19]. A recent meta-analysis found mortality risk to increase by 5 % for each additional hour spent sitting beyond seven hours per day [20]. Working adults who combine high levels of occupational sitting and high levels of TV viewing time are likely to accumulate at least seven hours of sitting across the day, suggesting a potential increased health risk for these workers. In addition, there is evidence to suggest that the adverse health effects of occupational sitting and leisure-time sitting combined may be greater than those associated with each behaviour separately [21].

Occupational sitting and TV viewing time may therefore be key areas to target in order to have the greatest impact in reducing overall levels of sedentary behaviour in working adults. Identifying attributes associated with high occupational sitting and also the combination of high occupational sitting with high TV viewing time is therefore likely to be important for informing intervention strategies, by determining which groups within working populations may benefit from targeted approaches to reduce both of these behaviours. Whether the factors influencing sitting time in these areas differ for women and men is also of interest, as gender differences have been reported in the correlates of both occupational sitting [13] and TV viewing time [22]. Notably, few studies have explored these potential variations, which may be important for understanding why some studies have found higher levels of occupational sitting amongst men compared with women [10, 12]. Further research in this area is warranted.

We first examined the socio-demographic and health-related correlates of high occupational sitting time compared to low occupational sitting time for women and men; and second, identified the correlates of having high occupational sitting time and high TV viewing time compared to low occupational sitting time and low TV viewing time, in a large sample of Australian women and men.

## Methods

### Participants & procedures

The Australian Diabetes, Obesity and Lifestyle study (AusDiab) is a national longitudinal study, designed originally to examine the prevalence and incidence of diabetes and its precursors in a population-based sample of Australian adults. Details of the data collection methods and response rates have been described previously [23]. Briefly, 11,247 adults participated in the baseline survey in 1999–2000. Follow-up studies were conducted in 2004–05 (AusDiab2) and 2011–12 (AusDiab3) with 6,400 and 4,614 participants completing follow-up (including biomedical examination) for AusDiab2 and AusDiab3 respectively [24]. The present study uses data from AusDiab3 and includes those participants who reported working or volunteering ≥ 35 h/week across weekdays (n = 1,378). Participants were excluded if they were pregnant (n = 2) or were missing data on any of the covariates of interest (n = 141). The final sample comprised 1,235 participants (466 women and 769 men). The Alfred Hospital Ethics Committee approved the study and written informed consent was obtained from all participants.

### Measures

#### Occupational sitting time and television (TV) viewing time

Occupational sitting time on weekdays and weekends was assessed by the question *“Please estimate the total time during the last week that you spent sitting down as part of your job while at work or working from home, including meal and snack breaks, sitting to do work such as at desk or in meetings, sitting to use the computer at work, and sitting for travel as part of work such as being a taxi driver?”*. Participants were asked to estimate the total time sitting first for Monday to Friday, and then for Saturday and Sunday. A similar question has previously been validated in a working adult population [25]. Modifications were made to this question to align with the AusDiab study format, including adding examples of occupational sitting behaviour. For this study, only weekday occupational sitting time was considered in analyses, as the majority of participants reported working zero hours across the weekend and only 6 % reported working for 15 h or more across the two weekend days. The average weekday occupational sitting time (hours/day) was calculated by dividing the reported hours participants sat for work on Monday-Friday by five. Participants were classified as having either high (>6 h/day) or low (≤6 h/day) weekday occupational sitting time based on a median split.

Television viewing time was collected from the question, *“Please estimate the total time during the last week that you spent watching TV or videos/DVDs. This is when it was the main activity that you were doing; for example you would not include time when the television was switched on and you were preparing a meal”*. This question has been shown to have established reliability and validity [26]. Average daily TV viewing time (hours/day) was calculated by adding weekday and weekend hours and dividing by seven. Participants were classified as having either high (≥1.5 h/day) or low (<1.5 h/day) TV viewing time based on a median split.

#### Socio-demographic attributes

Socio-demographic attributes, including gender and age, were determined from interviewer-administered questionnaires. Educational attainment (collected at baseline in 1999/2000) was categorised as high school or lower; technical/vocational (including trade or technician’s certificate, associate or undergraduate diploma, or nursing or teaching qualification); and, bachelor’s degree or higher. Occupation was collapsed from eight categories to three: *professional/managerial* (professionals, managers,); *white collar/administrative* (community & personal service workers, clerical & administrative workers, sales workers); *blue collar* (technicians & trades workers, machinery operators & drivers, labourers). Marital status was categorised as *married/de facto; separated/divorced/widowed; never married*. Presence of children in the household was categorised as yes or no. Annual gross household income was categorised into four categories: *less than $60,000*; *$60,000-$125,000*; *$125,000+*; *don’t know/preferred not to say*. Participants reported the number of hours and minutes they worked during the previous week on Monday to Friday. The average hours worked per weekday was calculated by dividing the total reported time by five.

#### Health-related attributes

Leisure-time physical activity (LTPA) was assessed using the Active Australia Survey Questionnaire, which assesses walking for recreation or transport, other moderate-intensity activity and vigorous-intensity activity [27]. LTPA was measured in minutes per week and participants were classified as either meeting (≥150 min/week), or not meeting (<150 min/week), adult physical activity guidelines [28]. Smoking status was categorised as current smoker, ex-smoker or non-smoker. Daily energy and alcohol intake were assessed through a self-administered food-frequency questionnaire [29]. Participants reported frequency of consumption of various food items, with the last 12 months as a reference. Gender-specific standard portion sizes were derived from weighed food records and the reported frequencies were converted to daily equivalents. NUTTAB95 food composition data was used to calculate the intake of energy [30]. Alcohol intake was measured in grams, and categorised as ≤10 g/day; >10- ≤ 20 g/day and >20 g/day, based on Australian National Health and Medical Research Council (NHMRC) guidelines [31] that suggest that adults drink no more than two standard drinks (20 g alcohol) on any day to limit long-term risk of alcohol-related harm.

Height and weight measurements were taken by trained AusDiab personnel at designated testing sites. BMI was calculated using the formula: weight (kg)/height (m)^2^ and categorised as underweight (<18.5), normal (18.5- < 25), overweight (25- < 30) or obese (≥30) [32]. Due to the small number of participants (<1 %) in the underweight category, the underweight and normal categories were combined.

### Statistical analyses

Dichotomous high/low categories of occupational sitting time and TV viewing time were used as the outcome variables in analyses. An *a priori* decision was made to stratify all regression analyses by gender. For the first aim, logistic regression analyses were conducted to identify socio-demographic and health-related correlates of high occupational sitting time. Univariate logistic regression models were first conducted to examine relationships between socio-demographic and health-related factors with the outcome variable (high vs low occupational sitting time). All available socio-demographic and health-related factors were then entered into the second set of logistic regression models. As these analyses were exploratory in nature, a backward regression approach was then applied, removing variables until only those significant at *p* < 0.20 remained, to achieve a parsimonious model. Age, average hours worked/day and LTPA were forced into all models. As there were only a small number of women in blue collar occupations who reported high occupational sitting, occupational status was changed to missing for these participants in the regression analyses, with comparisons made between the other two occupational groupings (white collar/administrative and professional/managerial).

To address the second aim, participants were grouped into one of four categories based on combinations of high or low for occupational sitting and TV viewing time. Multinomial logistic regression analyses, stratified by gender, were conducted to identify the socio-demographic and health-related correlates of being in each of the groupings with at least one ‘high’ category (low occupational sitting/high TV; high occupational sitting/low TV; high occupational sitting/high TV), compared with the category considered to be the lowest risk – the low occupational sitting/low TV viewing grouping (reference group). The same backward, stepwise regression approach described above was applied to achieve a parsimonious model. Analyses were conducted using Stata 12 for Windows (StataCorp, College Station, TX). Statistical significance was set at *p* < 0.05.

## Results

### Participant characteristics

The mean (SD) age of participants was 53 (7) years and 38 % were women (Table 1). There were significant differences between women and men in a number of socio-demographic and health-related attributes. Of note, a higher proportion of men had a technical/vocational level of educational attainment (48 % vs 35 %) and more were in blue collar occupations (31 % vs 6 %). A higher proportion of women were separated, divorced or widowed (20 % vs 7 %), worked in white collar/administrative occupations (40 % vs 11 %) and reported household incomes < $60,000 (21 % vs 13 %) compared with men. Men reported an additional one hour of LTPA per week, and higher energy intake and alcohol consumption than women.

**Table 1:** Participant characteristics by gender (mean (SD), % or median (IQR))

	Total sample (*n* = 1,235)	Women (*n* = 466)	Men (*n* = 769)	*p*
Socio-demographic attributes				
Age (years)	53.3 (7.2)	52.9 (6.8)	53.6 (7.4)	0.076
Education				*p* < 0.001
*High school or less*	24.4	30.5	20.7	
*Technical/vocational*	43.1	34.6	48.2	
*Bachelor’s degree or higher*	32.6	35.0	31.1	
Marital status				*p* < 0.001
*De facto/married*	81.9	72.5	87.7	
*Separated/divorced/widowed*	11.9	20.2	6.9	
*Single*	6.2	7.3	5.5	
Child(ren) in the household				
*Yes, %*	48.1	45.3	49.8	0.123
Occupation				*p* < 0.001
*Professional/managerial*	56.6	53.7	58.4	
*White collar/administrative*	21.9	40.1	10.9	
*Blue collar*	21.5	6.2	30.7	
Annual gross household income				*p* < 0.001
*Less than $60,000*	16.0	21.2	12.9	
*$60,000-$125,000*	40.4	37.8	42.0	
*$125,000+*	39.9	36.3	42.1	
*Don’t know/Preferred not to say*	3.6	4.7	3.0	
Average weekday hours worked	8.0 (8.0, 10.0)	8.0 (7.6, 9.0)	8.4 (8.0, 10.0)	*p* < 0.001
Health-related factors				
Leisure-time physical activity (min/week) - median (IQR)	240 (90, 500)	210 (60, 420)	270 (95, 540)	0.004
Physical activity guidelines				0.068
*Sufficiently active, %*	64.8	61.6	66.7	
*Insufficiently active, %*	35.2	38.4	33.3	
Smoking status				0.055
*Current smoker, %*	6.2	4.3	7.3	
*Ex-smoker, %*	32.4	31.1	33.2	
*Non-smoker, %*	61.5	64.6	59.6	
BMI (kg/m^2^)	27.9 (5.0)	27.5 (5.7)	28.2 (4.4)	0.034
*Normal, %*	29.4	39.1	23.5	*p* < 0.001
*Overweight, %*	43.6	32.4	50.3	
*Obese, %*	27.0	28.5	26.1	
Energy intake (kJ/day)	7036.5 (5367.8, 9091.6)	5661.3 (4552.1, 7014.3)	8083.1 (6370.9, 9989.7)	*p* < 0.001
Alcohol consumption (g/day)	9.7 (2.1, 23.6)	5.3 (1.2, 15.1)	13.7 (3.2, 30.0)	*p* < 0.001
*≤10*	50.9	64.8	42.4	
*>10- ≤ 20*	18.0	16.7	18.7	
*>20*	31.2	18.5	38.9	
Sitting time				
Weekday work sitting time (hours/day)	6.0 (3.0, 7.6)	6.0 (2.8, 7.5)	6.0 (3.0, 7.6)	0.924
TV viewing time (hours/day)	1.4 (0.7, 2.1)	1.3 (0.7, 2.0)	1.6 (0.9, 2.3)	*p* < 0.001

### Correlates of high occupational sitting time

The socio-demographic and health-related correlates of high occupational sitting time (reference: low occupational sitting time), stratified by gender, are shown in Table 2. In the fully adjusted models for women (adjusted for age, hours worked, TV viewing time, LTPA and all other remaining covariates), higher household income remained the strongest correlate: the odds of being in the high occupational sitting group increased over twofold for women with household incomes of $60,000-$125,000 and $125,000+ respectively, compared with those on less than $60,000. Women who were separated, divorced or widowed (compared with being de facto or married) were nearly twice as likely to have high levels of occupational sitting. In addition, the odds of being in the high occupational sitting group decreased slightly with age.

**Table 2:** Socio-demographic attributes and health-related factors associated with high occupational sitting time compared with low occupational sitting time: stratified by gender

	Women				Men			
Correlates	Unadjusted odds ratio (95 % CI)	*p*	Fully adjusted odds ratio (95 % CI)^ab^	*p*	Unadjusted odds ratio (95 % CI)	*p*	Fully adjusted odds ratio (95 % CI)^ab^	*p*
Socio-demographic attributes								
Age (years)	0.96 (0.93, 0.99)**	0.003	0.96 (0.94, 0.99)*	0.021	0.98 (0.96, 1.00)	0.096	0.99 (0.97, 1.02)	0.578
Educational attainment								
*High school or less*	1.00		-		1.00		1.00	
*Technical/vocational*	0.96 (0.60, 1.52)	0.858	-		0.54 (0.36, 0.79)**	0.002	0.58 (0.38, 0.88)*	0.011
*Bachelor’s degree or higher*	1.51 (0.96, 2.38)	0.076	-		1.77 (1.18, 2.65)**	0.006	1.10 (0.68, 1.78)	0.711
Marital status								
*De facto/married*	1.00		1.00		1.00		-	
*Separated/divorced/ widowed*	1.19 (0.75, 1.89)	0.453	1.99 (1.15, 3.46)*	0.014	0.55 (0.29, 1.01)	0.054	-	
*Single*	1.79 (0.88, 3.65)	0.108	2.01 (0.92, 4.43)	0.082	0.55 (0.28, 1.10)	0.091	-	
Child(ren) in the household	0.98 (0.68, 1.42)	0.931	-		1.67 (1.25, 2.23)**	0.001	1.31 (0.93, 1.83)	0.124
Occupation								
*Professional/ managerial*	1.00		-		1.00		1.00	
*White collar/ administrative*	0.99 (0.68, 1.45)	0.976	-		0.74 (0.46, 1.18)	0.201	1.12 (0.66, 1.90)	0.683
*Blue collar*	^c^		-		0.20 (0.13, 0.29)***	*p* < 0.001	0.28 (0.18, 0.45)***	*p* < 0.001
Annual gross household income								
*Less than $60,000*	1.00		1.00		1.00		1.00	
*$60,000-$125,000*	2.30 (1.37, 3.87)**	0.002	2.71 (1.53, 4.79)**	0.001	2.49 (1.44, 4.31)**	0.001	1.62 (0.89, 2.93)	0.113
*$125,000+*	2.02 (1.19, 3.41) **	0.009	2.61 (1.38, 4.95)**	0.003	4.37 (2.53, 7.54)***	*p* < 0.001	1.86 (1.00, 3.45)*^f^	0.049
*Don’t know/ Preferred not to say*	0.68 (0.23, 2.00)	0.480	1.04 (0.33, 3.31)	0.944	1.49 (0.52, 4.27)	0.462	0.77 (0.25, 2.41)	0.656
Average weekday hours worked	1.10 (0.97, 1.25)	0.130	1.11 (0.98, 1.27)	0.107	1.28 (1.17, 1.40)***	*p* < 0.001	1.26 (1.14, 1.39)***	*p* < 0.001
Health-related factors								
Leisure-time physical activity (mins/week)^d^	0.99 (0.98, 1.01)	0.442	0.99 (0.97, 1.01)	0.269	0.99 (0.98, 1.00)	0.123	0.98 (0.97, 1.00)*^f^	0.014
Smoking status								
*Current smoker*	1.00		-		1.00		-	
*Ex-smoker*	1.03 (0.40, 2.67)	0.953	-		2.07 (1.07, 3.98)*	0.030	-	
*Non-smoker*	1.22 (0.48, 3.07)	0.673	-		2.17 (1.15, 4.08)*	0.017	-	
BMI (kg/m^2^)	1.01 (0.98, 1.04)	0.586	-		1.02 (0.99, 1.05)	0.208	-	
Energy intake (kJ/day)^e^	1.00 (0.99, 1.01)	0.414	-		0.99 (0.99, 1.00)* ^f^	0.028	-	
Alcohol consumption (g/day)								
*≤10*	1.00		1.00		1.00		-	
*>10- ≤ 20*	1.63 (0.99, 2.70)	0.054	1.53 (0.91, 2.57)	0.108	0.86 (0.58, 1.29)	0.474	-	
*>20*	1.22 (0.76, 1.98)	0.411	1.11 (0.67, 1.83)	0.693	0.92 (0.67, 1.27)	0.626	-	
Sitting time								
TV viewing time (average hours/day)	0.99 (0.82, 1.20)	0.937	1.06 (0.86, 1.29)	0.601	0.87 (0.76, 1.00)	0.052	1.00 (0.86, 1.17)	0.958

Women: low work sitting category (*n* = 264); high work sitting category (*n* = 202). Men: low work sitting category (*n* = 459); high work sitting category (*n* = 310)

*p < 0.05 **p < 0.01 ***p < 0.001

^a^Logistic regression model for women adjusted for age, marital status, household income, average weekday hours worked, alcohol consumption, leisure-time physical activity and TV viewing time

^b^Logistic regression model for men adjusted for age, educational attainment, children in the household, occupation, household income, average weekday hours worked, leisure-time physical activity and TV viewing time

^c^Women in blue collar occupations were excluded from this analysis due to the small number of blue collar workers in the high occupational sitting group

^d^OR corresponds to each additional 30 min/week of leisure-time physical activity

^e^OR corresponds to each additional 100 kJ of energy consumed per day

^f^Significant confidence intervals include the value of 1.00 due to rounding

In the fully adjusted models for men, educational attainment, occupation and household income remained significant, although the association of household income with high occupational sitting was diminished. Being in a blue collar occupation and having a technical or vocational education were associated with lower odds of being in the high occupational sitting group, compared with their respective comparison categories. Each one hour increase in work hours was associated with 26 % higher odds of being in the high occupational sitting group, while each 30 min increase in leisure-time physical activity per week was associated with a small, but significant decrease in the odds of men being in the high occupational sitting group.

### Correlates of high occupational sitting and high TV viewing time

The results of the multinomial logistic regression analyses are shown in Table 3 (women) and Table 4 (men). Age, hours worked and leisure-time physical activity were adjusted for in both models and the low occupational sitting/low TV viewing category was the reference category for all comparisons.

**Table 3:** Associations of socio-demographic and health-related factors with occupational sitting/TV viewing time categories – women

	Low occupational sitting/High TV viewing time (*n* = 111)		High occupational sitting/Low TV viewing time (*n* = 127)		High occupational sitting/High TV viewing time (*n* = 75)	
Correlates	RRR (95 % CI)	*p*	RRR (95 % CI)	*p*	RRR (95 % CI)	*p*
Socio-demographic						
Age	1.07 (1.03, 1.12)**	0.001	0.97 (0.94, 1.01)	0.190	1.02 (0.97, 1.07)	0.430
Marital status						
*De-facto/married*	1.00		1.00		1.00	
*Separated/divorced/ widowed*	0.47 (0.22, 0.99)*	0.048	1.35 (0.68, 2.70)	0.393	1.58 (0.72, 3.47)	0.252
*Single*	1.87 (0.59, 5.95)	0.288	1.92 (0.63, 5.85)	0.253	3.89 (1.23, 12.26)*	0.021
Annual gross household income						
*Less than $60,000*	1.00		1.00		1.00	
*$60,000-$125,000*	0.85 (0.42, 1.70)	0.648	2.52 (1.19, 5.33)*	0.015	2.85 (1.26, 6.49)*	0.012
*$125,000+*	1.04 (0.48, 2.22)	0.924	2.83 (1.24, 6.49)*	0.014	2.51 (0.96, 6.56)	0.062
*Don’t know/ Preferred not to say*	0.83 (0.26, 2.68)	0.759	0.86 (0.19, 3.87)	0.842	1.22 (0.21, 6.99)	0.821
Average weekday hours worked	0.91 (0.76, 1.10)	0.346	1.15 (0.99, 1.35)	0.075	0.91 (0.72, 1.14)	0.416
Health-related						
BMI	1.03 (0.99, 1.08)	0.141	1.00 (0.96, 1.05)	0.965	1.04 (0.99, 1.09)	0.107
Leisure-time physical activity (mins/week)^a^	0.98 (0.96, 1.00)	0.073	0.99 (0.97, 1.01)	0.297	0.97 (0.94, 1.00)*^c^	0.040
Energy consumption (kJ/day)^b^	1.01 (1.00, 1.02)	0.108	1.00 (0.99, 1.02)	0.696	1.02 (1.00, 1.03)*^c^	0.024

Multinomial logistic regression model: reference group is low occupational sitting time, low TV viewing time (*n* = 153)

Multinomial logistic regression model results presented are adjusted for all other variables included in the table

RRR: Relative risk ratio; CI: confidence interval

*p < 0.05 **p < 0.01

^a^RRR corresponds to each additional 30 min/week of leisure-time physical activity

^b^RRR corresponds to each additional 100 kJ of energy consumed per day

^c^Significant confidence intervals include the value of 1.00 due to rounding

**Table 4:** Associations of socio-demographic and health-related factors with occupational sitting/TV viewing time categories – men

	Low occupational sitting/ High TV viewing time (n = 256)		High occupational sitting/ Low TV viewing time (n = 162)		High occupational sitting/ High TV viewing time (n = 148)	
Correlates	RRR (95 % CI)	*p*	RRR (95 % CI)	*p*	RRR (95 % CI)	*p*
Socio-demographic						
Age	1.04 (1.01, 1.07)**	0.004	1.02 (0.99, 1.05)	0.262	1.01 (0.98, 1.04)	0.472
Educational attainment						
*High school or less*	1.00		1.00		1.00	
*Technical/vocational*	1.06 (0.64, 1.77)	0.812	0.68 (0.36, 1.26)	0.218	0.55 (0.30, 0.99)*	0.045
*Bachelor’s degree or higher*	0.58 (0.30, 1.12)	0.103	1.06 (0.54, 2.09)	0.869	0.72 (0.37, 1.41)	0.341
Child at home	1.41 (0.92, 2.16)	0.110	2.20 (1.36, 3.55)**	0.001	1.17 (0.73, 1.89)	0.519
Occupation						
*Professional/managerial*	1.00		1.00		1.00	
*White collar/ administrative*	2.69 (1.29, 5.60)**	0.008	1.74 (0.75, 4.06)	0.198	2.31 (1.05, 5.07)*	0.037
*Blue collar*	1.76 (1.08, 2.86)*	0.022	0.36 (0.18, 0.69)**	0.002	0.42 (0.23, 0.80)**	0.008
Annual gross household income						
*Less than $60,000*	1.00		1.00		1.00	
*$60,000-$125,000*	1.12 (0.63, 1.98)	0.694	3.30 (1.17, 9.32)*	0.024	1.21 (0.57, 2.57)	0.618
*$125,000+*	1.57 (0.83, 2.98)	0.162	3.76 (1.30, 10.88)*	0.015	1.90 (0.86, 4.19)	0.114
*Don’t know/Preferred not to say*	2.57 (0.76, 8.66)	0.127	2.29 (0.39, 13.58)	0.363	1.09 (0.22, 5.46)	0.916
Average weekday hours worked	0.82 (0.72, 0.94)**	0.005	1.27 (1.12, 1.45)***	*p* < 0.001	1.00 (0.87, 1.15)	0.991
Health-related						
BMI	1.05 (1.01, 1.10)*	0.024	1.03 (0.98, 1.09)	0.252	1.05 (1.00, 1.11)*^c^	0.045
Leisure-time physical activity (mins/week)^a^	0.98 (0.97, 1.00)*^c^	0.022	0.98 (0.96, 1.00)* ^c^	0.014	0.97 (0.96, 0.99)**	0.003
Energy consumption (kJ/day)^b^	1.01 (1.00, 1.01)	0.106	0.99 (0.99, 1.00)	0.220	1.01 (1.00, 1.01)	0.209

Multinomial logistic regression model: reference group is low occupational sitting time, low TV viewing time (*n* = 203)

Multinomial logistic regression model results presented are adjusted for all other variables included in the table

RRR: Relative Risk Ratio; CI: confidence interval

*p < 0.05 **p < 0.01 ***p < 0.001

^a^RRR corresponds to each additional 30 min/week of leisure time physical activity

^b^RRR corresponds to each additional 100 kJ of energy consumed per day

^c^Significant confidence intervals include the value of 1.00 due to rounding

For women (Table 3), socio-demographic attributes associated with being in the high occupational sitting/high TV viewing group included marital status and income. Single women, relative to de facto/married women, had a higher risk of being in the high occupational sitting/high TV group than the low occupational sitting/low TV viewing group, although the wide confidence interval suggests some degree of uncertainty with this finding. Having a household income of $60,000-$125,000 (ref < $60,000) was associated with a nearly three times higher relative risk ratio of being in the high occupational sitting/high TV group compared to the low occupational sitting/low TV group. Of the health-related factors, energy consumption was positively associated, while leisure-time physical activity was negatively associated, with being in the high occupational sitting/high TV viewing time group relative to the low occupational sitting/low TV viewing group, although effect sizes were small.

The factors identified above, apart from household income, were associated with the high occupational sitting/high TV viewing category only and not either of the other two occupational sitting/TV viewing categories. In contrast, higher household income was also associated with higher risk of being in the high occupational sitting/low TV viewing category compared to the low occupational sitting/low TV viewing group. Age was positively associated with being in the low occupational sitting and high TV viewing, relative to the low occupational sitting/low TV viewing group, but no significant association was observed between age and the high occupational sitting groups.

For men (Table 4), occupation was a significant correlate of combined high levels of occupational sitting and TV viewing time. Compared to men in managerial/professional occupations, men in blue collar occupations were less likely to be in the high occupational sitting/high TV viewing group than the low occupational sitting/low TV viewing group, while men in white collar/administrative jobs were more likely. Having a technical/vocational level of educational attainment (ref: high school or less) was associated with a lower relative risk of being in the high occupational sitting/high TV viewing group compared to the low occupational sitting/low TV viewing group. Of the health-related factors, higher levels of LTPA were associated with reduced risk of high occupational sitting/high TV viewing, and a lower risk of being in each of the other two high sitting groups (low occupational sitting/high TV, high occupational sitting/low TV), compared to the low occupational sitting/low TV viewing group. As BMI increased, there was a corresponding increase in the relative risk of being in the high occupational sitting/high TV group, as well as the other high TV category (low occupational sitting/high TV group) compared to the low occupational sitting/low TV viewing group

Attributes associated with the other two occupational sitting/TV viewing categories for men included occupation, income, hours worked and having a child at home. Compared with men in managerial/professional occupations, blue collar workers were also less likely to be in the other high occupational sitting group (high occupational sitting/low TV viewing) while white collar workers were more likely to be in the other high TV viewing group (low occupational sitting/high TV viewing), compared to the low occupational sitting/low TV viewing group. Income had a positive association with being in the high occupational sitting/low TV group only (although confidence intervals were wide), while having a child at home was also associated with increased likelihood of being in this group, compared to the low occupational sitting/low TV viewing group. An increase in hours worked per day was associated with a lower risk of being in the low occupational sitting/high TV viewing group and a higher risk of being in the high occupational sitting/low TV viewing group, compared to the low occupational sitting/low TV viewing group.

## Discussion

Research on the correlates of occupational sedentary behaviour is still in its infancy, despite growing interest in workplace-based initiatives to address excessive sitting time. In this sample of full-time Australian workers, we observed variations between women and men in the attributes associated with high occupational sitting, and high occupational sitting and TV viewing time in combination.

### Correlates of high occupational sitting time

Of the socio-demographic attributes, household income was the strongest correlate of high occupational sitting in both women and men. This is consistent with other studies [10, 13] and is likely to reflect the tendency for many higher paid jobs to be office-based. For women, the only other significant correlates were age and marital status. Separated/divorced or widowed participants were found to be more likely to be in the high occupational sitting category than married/de facto women and the odds of having high occupational sitting decreased with age. Others have also reported a similar finding of lower levels of occupational sitting with increasing age [13, 33], although the reasons for this association are unclear. As our models controlled for the number of hours worked it appears unlikely that this is due to older people working fewer hours.

Other factors were identified as correlates amongst men only. Similar to previous findings [11, 12], men employed in white collar or managerial/professional occupations were more likely to have higher levels of occupational sitting than blue collar workers. Considering the tasks and roles performed by these occupational groups – which are likely to be office-based – this is not overly surprising. The small number of women employed in blue collar occupations precluded exploration of whether this association also holds for women in our sample, but others have confirmed this association amongst women in an Australian population [12].

### Correlates of high occupational sitting and high TV viewing time

In line with the identified correlates of high occupational sitting on its own, higher household income was associated with an increased likelihood of being in each of the two high occupational sitting groups for women, relative to the low occupational sitting, low TV viewing group. Interestingly, single women were more likely to have high levels of both occupational sitting and TV viewing time than married/de facto women, which may be due to fewer domestic responsibilities. Few studies have explored the association between marital status and sedentary behaviours by gender. One previous study found support for higher levels of TV viewing amongst single women compared to women who were married or in de facto relationships [34], however another [22] found no significant differences by marital status. In a sample of working adults of both genders Clemes et al. [35] found higher daily sitting times on workdays for those who were single, divorced or widowed, compared with those who were married/de facto, including higher levels of sedentary leisure activities. However, no differences were observed for sitting time at work.

Amongst men, blue collar workers (compared with managerial/professional workers) were more likely to be in the high TV viewing categories and less likely to be in the high occupational sitting/low TV viewing category. Workers in manual jobs tend to have higher levels of occupational physical activity than white collar or professional workers [36, 37] which could suggest a compensatory effect. However, previous studies have generally found no difference in leisure-time sitting between those with high and low occupational sitting time [11, 38, 39] and Chau et al. [40] found that workers in physically demanding/heavy labour occupations were less likely to have high levels of leisure-time sitting. Alternatively, occupational category may be a proxy measure for socioeconomic position in this sample; people in lower socioeconomic groupings have been found to spend more time watching TV [41].

Certain health-related factors were also associated with higher levels of occupational sitting and TV viewing time, which is broadly consistent with what has been reported by previous studies [42, 43] suggesting that high levels of sitting may occur alongside other unhealthy behaviours. For women, energy intake was positively associated with being in the “high risk”, high occupational sitting/high TV group compared with the low occupational sitting/low TV viewing group. For men BMI was positively associated with being in both high TV viewing groups (combined with both low and high occupational sitting), suggesting that this association may be more of a reflection of the levels of TV viewing than the high occupational sitting. Higher levels of TV viewing time have previously been found to be associated with higher consumption of high energy snack foods [44] and increased risk of obesity [19, 45]. It is of interest however, that no association was observed between high sitting time and BMI for women, in light of the higher energy intake for those in the high occupational sitting, high TV viewing group.

Leisure-time physical activity (LTPA) appeared to be more strongly associated with occupational sitting time amongst men than women. For men, higher levels of LTPA were associated with a lower likelihood of high occupational sitting, and being in each of the three high categories of occupational sitting/TV viewing. For women, higher physical activity levels were significantly associated with a lower risk of being in the high occupational sitting/high TV category, but the magnitude of the association was small. We found no evidence to support a ‘compensation’ effect– whereby participants with high levels of workplace sitting undertake more physical activity in their leisure time [40]. In contrast, it appears that those who were engaged in the lowest levels of sitting during the day, particularly for work, were also more likely to be active during leisure time and this association was observed to be stronger for men than women. Studies using both objective and self-report measures of sitting time have reported weak correlations between LTPA and sedentary behaviour [5, 46]; however these generally have not been stratified by gender. Further research is needed to explore potential associations between occupational and leisure sitting time with LTPA, including separately for women and men.

The observation that a number of health-related correlates were associated with being in the group with high occupational sitting and high TV viewing suggests that an intervention that also includes elements targeting other health behaviours (e.g. healthy eating; promotion of leisure-time physical activity) in conjunction with efforts to reduce sedentary behaviour may be of benefit to those with high levels of occupational sitting time and low levels of occupational physical activity. The workplace has previously been identified as a key target setting for implementing health promotion interventions more generally, with workplace interventions found to be beneficial for increasing physical activity, improving fitness levels and reducing diabetes risk [47, 48]. Knowledge gained from previous successful programs that have targeted, for example, physical activity and healthy food choices, may be useful for the design of workplace interventions to reduce sedentary behaviour. To ensure maximum impact, it will be important to ensure that such programs capture those most at risk (e.g. high levels of occupational sitting and high TV viewing time with low levels of leisure-time physical activity and poor diet quality).

Further research is needed to identify potentially modifiable environmental and social correlates of occupational sitting. Our findings were in line with previous research [10–13] indicating that work-related factors – occupation and income levels – were correlates of high levels of occupational sitting time. As such, exploration of the relative influence of the workplace environment and broader workplace culture is likely to be beneficial, as they may be key drivers of sedentary behaviour [49]. This could include studying organisational strategies common in some office-based organisations such as job rotation, hot-desk arrangements and flexible working patterns. In the context of increasing interest in the effectiveness and feasibility of implementing activity-permissive work practices in the office environment [50], there is a need for high quality evidence on the multiple individual and interacting influences on occupational sedentary behaviour.

Strengths of this study include the large sample of workers from a range of backgrounds, located across urban and regional areas of Australia. The analysis of a range of potential socio-demographic and health-related correlates of both occupational sitting and TV viewing time is also an important contribution. However, there are some limitations. While participants in AusDiab were originally recruited as a population-based sample, those who participated in the 2011/12 follow-up were younger, less likely to live in a socioeconomically disadvantaged area, had a higher level of education and lower BMI than those who didn’t participate [51]. This is similar to factors relating to attrition in another large Australian longitudinal survey [52] and may have introduced bias into our results. As this was a 12 year follow up, participants were also generally older (median age 53), with limited representation of younger workers. These factors should be taken into consideration when interpreting our findings. Investigating whether patterns of sedentary behaviour differ in younger (i.e. less than 35 years) and older workers would be beneficial as their experiences with technology and work environments are likely to differ.

Another potential limitation is that occupational sitting time and TV viewing time were self-reported. Self-report measures permit investigation of sitting in particular domains (e.g. work, leisure), which was of interest to this study. However, the reliance on self-report may have introduced recall error, including possible misclassification of the outcome measure. While a number of self-report questions on occupational sitting time and TV viewing time, such as the ones used in the present study, have previously been validated and considered to be acceptable for use in population-level studies [15, 25], it has been suggested that they may not be highly accurate on an individual level, particularly for low and high levels of sitting [15, 25, 53]. However, as these measures were used in this study to categorise participants into dichotomous low/high categories, misclassification of the outcome is expected to be minimal. The nature of the occupational sitting time question used in this cohort study also precluded examination of patterns of sitting amongst adults who work non-standard weeks, for example, shift workers or casual workers. Consequently, assumptions were made that participants worked a similar number of hours across each weekday. Further studies should seek to explore correlates of sitting amongst workers from a range of different working patterns. Objective measurement of sitting time combined with the use of self-report diaries or location sensors could enable more accurate measurement of occupational sitting time, including capturing the time of day for both work hours and sitting time and the length of time spent in prolonged bouts of sitting. Furthermore, the cross-sectional design of this study precludes inferences regarding causality, restricting analysis to correlates, rather than determinants of sitting time.

## Conclusions

Socio-demographic attributes (higher household income; being separated, divorced or widowed; and younger age amongst women; professional/managerial occupation and higher educational attainment amongst men), were identified as correlates of high occupational sitting time, while certain health-related factors (lower leisure-time physical activity; higher BMI amongst men, higher energy consumption amongst women) were also associated with high levels of occupational sitting and TV viewing in combination. As some of the attributes associated with high occupational sitting, and high occupational sitting/high TV viewing time differed between women and men, targeted sitting time reduction strategies according to gender may need to be considered. Building this evidence base on occupational sedentary behaviour will assist in the development of approaches needed to address an emerging work health and safety issue.

### Availability of data and materials

Not applicable
